# Alcohol Consumption Accumulation of Monocyte Derived Macrophages in Female Mice Liver Is Interferon Alpha Receptor Dependent

**DOI:** 10.3389/fimmu.2021.663548

**Published:** 2021-04-30

**Authors:** Khaled Alharshawi, Holger Fey, Alyx Vogle, Tori Klenk, Miran Kim, Costica Aloman

**Affiliations:** Division of Digestive Diseases and Nutrition, Section of Hepatology, Rush University, Chicago, IL, United States

**Keywords:** monocytes, alcohol (EtOH), liver injury, mouse, female, male, Meadows-Cook model (MC)

## Abstract

Monocytes develop in the bone marrow from the hematopoietic stem cells and represent heterogeneous phagocyte cells in the circulation. In homeostatic and inflammatory conditions, after recruitment into tissues, monocytes differentiate into macrophages and dendritic cells. Alcohol use causes about 3.3 million worldwide deaths per year, which is about 5.9% of all deaths. In the United States and Europe, alcohol use disorders represent the fifth leading cause of death. Females are more susceptible to alcoholic liver injury in both humans and mice. Strikingly, we still do not know how much of this difference in tissue injury is due to the differential effect of alcohol and its toxic metabolites on a) parenchymal or resident cells and/or b) immune response to alcohol. Therefore, we used a model of chronic alcohol exposure in mice to investigate the dynamics of monocytes, an innate immune cell type showed to be critical in alcoholic liver injury, by using immunophenotypic characterization. Our data reveal a sex-dimorphism of alcohol response of hepatic monocytes in female mice that is interferon receptor alpha dependent. This dimorphism could shed light on potential cellular mechanism(s) to explain the susceptibility of females to alcoholic immunopathogenesis and suggests an additional targetable pathway for alcoholic liver injury in females.

## Introduction

Monocytes originate in the bone marrow (BM) from the hematopoietic stem cells and represent a heterogeneous population of phagocytes in the circulation (1, 2). In homeostatic and inflammatory conditions, after recruitment into tissues, monocytes differentiate into macrophages and dendritic cells (DC) (1, 2). In both humans and mice, monocytes are classified into subsets based on differential expression of specific markers and function (3–5). In mice, monocytes are classified based on the expression of lymphocyte antigen 6 complex, locus C1 (Ly-6C), C-C motif chemokine receptor 2 (CCR2), and C-X3-C motif chemokine receptor 1 (CX3CR1) (1, 2). Monocytes expressing high levels of Ly-6C and CCR2 (Ly-6C^hi^CCR2^hi^) and rapidly migrate to sites of inflammation to give rise to pro-inflammatory macrophages and DCs (1, 2, 4, 5). The locally patrolling monocytes (pro-repair) express high levels of CX3CR1 but low levels of both Ly-6C and CCR2 and described phenotypically as Ly-6C^lo^CX3CR1^hi^ (1, 2, 4, 5).

Alcohol use accounts for about 3.3 million worldwide deaths annually, which is about 5.9% of all deaths (6). In the United States and Europe, alcohol use disorders represent the fifth leading cause of death (6). Alcohol consumption induces damage to multiple organs, including the liver, brain, gut, pancreas, and lungs (7, 8). Alcohol use induces tissue injury by a complex interaction between toxic effects of alcohol metabolites, including acetaldehyde, reactive oxygen, and nitrogen species and the impact of alcohol on the immune system (8, 9). The primary metabolism of alcohol occurs in the liver; hence it suffers the most significant damage due to alcohol consumption (7, 8).

Evidence in the literature indicates that alcohol induces increased intestinal permeability that allows the intestinal bacterial production, i.e. endotoxins or lipopolysaccharide (LPS), to reach the portal circulation (10–12) and Kupffer cells (KC), tissue-resident macrophages in the liver. Portal and systemic bacterial products have a well validated critical role in alcoholic tissue injury by recruitment and activation of the immune cells, histologically documented by the accumulation of inflammatory cells (10, 11). Innate immune cells such as neutrophils and Ly-6C^hi^ monocytes infiltrate liver tissues, are well defined participants in alcoholic tissue injury (6, 13, 14).

Mononuclear phagocytes, monocytes and macrophages, play a critical role in the pathogenesis of alcoholic liver disease (ALD) (15). KC represent the majority of liver macrophages in the steady-state (16). However, acute and chronic liver injury induces the recruitment of circulating monocytes into the liver, where they differentiate into macrophages and play a critical role in eliminating pathogens and induce tissue repair (16, 17).

The susceptibility of females to autoimmune diseases is well documented and was attributed at least partially to Interferon I signaling (18). Similarly, females are more susceptible to alcoholic liver injury in both humans and mice (19–21). Strikingly, we still do not know how much of this difference in susceptibility to alcohol tissue injury is due to the specific effect of alcohol and its toxic metabolites on a) parenchymal or resident hepatic cells and b) how much is due to sex specific immune response to alcohol (8, 9).

Therefore, we used a simple model of chronic alcohol exposure in mice to investigate the dynamics of monocytes, the most studied innate immune cell population, to alcohol exposure. Our data reveal a sex-dimorphism of alcohol response of hepatic monocyte-derived macrophages in female mice that is interferon receptor alpha dependent. This dimorphism sheds light on potential cellular mechanism(s) to explain the susceptibility of females to alcohol immunopathogenesis and suggest an additional targetable pathway for alcoholic liver injury in females.

## Materials and Methods

### Mice

Female and male Wild type (WT) C57BL/6 (stock # 000664) and Interferon α/β receptor 1-knockout (IFNR^KO^) (stock # 32045-JAX) mice were purchased from Jackson Labs, Sacramento, CA. Six-week-old mice received ethanol (EtOH) (Pharmaco, Greenfield Global, Brookfield, CT) in water as per Meadows-Cook model (MC) and normal chow *ad libitum* for four weeks. EtOH concentration increased from 0% to 20% (v/v) gradually (5%, 10% and 15% for 4 days interval between each change then up to 20% in the 3^rd^ week). Mice were placed on MC diet for 4 weeks. Liver and body weight were measured, and whole blood, BM, liver, spleen, and lungs were collected for analysis. All mice were kept in an infection-free environment, and only control mice had access to alcohol-free water. All experiments were approved by the Rush University Medical Center, Institutional Animal Care & Use Committee and performed following the guidelines of the National Institutes of Health.

### Leukocyte Isolation

Whole blood was obtained by cardiac puncture. After clot formation, samples were centrifuged and sera were obtained and used for analysis. Hepatic leukocyte isolation was performed as described previously (22) with minor modifications. Briefly, to eliminate circulating leukocytes in livers, each mouse was perfused with 10 mL of cold PBS (Fisher bioreagents, Pittsburgh, PA) *via* the portal vein. Lungs and livers were harvested and cut into small pieces (about 1 mm^2^) on ice then pushed through a 70 µm filter (Biologix group limited, Shandong, China). After washing, pellets were resuspended in 8 ml of 40% Percoll (GE Healthcare, Waukesha, WI) and layered on top of 3 ml of 70% Percoll and centrifuged at 900 x g for 25 minutes at room temperature. Leukocytes were collected from the interface of 40% and 70% Percoll for counting and staining. Splenocytes were collected by mashing the spleen through a 70 µm filter. BM cells were flushed with 10 mL of cold PBS from the femur and tibia. Red cell lysis of spleen and BM cells were performed before counting and staining.

### Flow Cytometry

Samples were stained with fixable viability dye eFluor 506 eBioscience (Thermo Fisher Scientific, Waltham, MA) and fluorophore-conjugated antibodies against mouse CD16/32, CD45, CD45R/B220, CD3, CD19, NK1.1, Ly-6C, Ly-6G, PDCA-1, MHC-II, CD11b, CD11c, CD103, CD115, CD117, CD127, CD135, F4/80, Gr-1, Ki-67, Sca-1, and TER-119 (**Supplementary Table 1**). Unless stated otherwise, all cell populations were gated as shown in **Supplementary Table 2**. Ki-67 relative mean fluorescent intensity (MFI) was calculated by dividing the MFI of each sample by the average MFI of the female control. Samples were acquired using an LSRFortessa flow cytometer (BD Bioscience, San Jose, CA). FlowJo software version 10.0.8r1 (FlowJo, LLC. Becton, Dickinson and Company, Franklin Lakes, NJ) was used for the analysis of the acquired flow cytometry data.

### ELISA

Blood was obtained by cardiac puncture at the time of euthanasia and centrifuged at 6,000 x g for 5 min for serum collection. Serum macrophage-colony stimulating factor (M-CSF) was measured by ELISA (MMC00, R&D systems, Minneapolis, MN). Serum alanine aminotransferase (ALT) and aspartate aminotransferase (AST) were measured using the ALT and AST Reagents (7526 and 7561, Pointe Scientific, Canton, MI). Serum LPS (endotoxins) was measured, according to manufacturer instruction, by PYROGENT-5000 Kinetic Turbidimetric LAL Assay (N383, Lonza, Morristown, NJ)

### Triglyceride Measurements

Hepatic triglycerides were measured using the triglyceride assay kit according to the protocol provided by the manufacturer (ab65336, Abcam, Cambridge, MA).

### Histology

#### Hematoxylin and Eosin (HE) Staining

For assessment of steatosis by histological analysis, the liver tissue was fixed in 10% formalin for 24 h, paraffin-embedded, sectioned at 5 μm and stained with hematoxylin and eosin.

#### Immunofluorescence

Cryo-sectioned 5 μm liver tissue slides were brought to room temperature and fixed with cold acetone for 8 min and then washed in PBS containing 0.05% Tween 20 (PBS-T). Nonspecific reactions were blocked with 5% normal goat serum (ab7481, Abcam, Cambridge, MA) and 5% TruStain fcX (BioLegend, San Diego, CA) in PBS-T for 1 h and then incubated with rabbit anti-mouse F4/80 (1:200, ab111101, Abcam) and rat anti-mouse Ly-6C (1:200, ab24973, Abcam) at 4°C overnight. After washing in PBS-T, the specimens were incubated with Alexa Fluor 488 goat anti-rat IgG (H+L) (1:400, #4416, Cell Signaling, Danvers, MA) and Alexa Fluor 555 goat anti-rabbit (1:400 #4413, Cell Signaling) for 1 hour at room temperature, washed again, treated with Vector TrueVIEW Autofluorescence Quenching Reagent and then counterstained with VECTASHIELD Mounting Medium with 4’,6-diamino-2-phenylindole (DAPI) (both: Vector Labs, Burlingame, CA). The Zeiss Axio Observer Microscope (Carl Zeiss Micro Imaging, Inc., Thornwood, NY) equipped with the Zen pro 2.3 software was used to visualize the immunofluorescence staining for F4/80 and Ly-6C, and nuclear localization was provided by DAPI. The negative controls were obtained by incubating sections with non-specific rat IgG or rabbit IgG as described above.

### qRT-PCR

Total hepatic mRNA was isolated from flash frozen liver in liquid nitrogen. The University of Illinois at Chicago (UIC) Genomics Research Core processed the specimens for total mRNA extraction, cDNA synthesis. qRT-PCR for cytokines, chemokines, and adhesion molecules implicated in monocyte trafficking was done, using primers described in **Supplementary Table 3**, on the ViiA 7 Real-Time PCR System (Applied Biosystems, Foster City, CA). Fold change was calculated using the comparative Ct method. Housekeeping genes used are beta-Actin (*Actb*) and beta-2-microglobulin (*B2m*).

### Statistical Analysis

All figures generated and all statistical analyses were done using GraphPad Prism version 8.3.0 (GraphPad Software, Inc. San Diego, CA). Two way ANOVA with multiple comparisons is used to calculate the p-values. A p-value of ≤ 0.05 was considered significant. The level of significance indicated by asterisks as follow: *p<0.05, **p<0.01, ***p<0.001, and ****p<0.0001. Unless stated otherwise, the data are presented as means ± standard error of the mean (SEM).

## Results

### Consumption of Alcohol Resulted in an Increase in Recruited Monocytes/Macrophages in the Livers of Female Mice

To investigate the effect of alcohol consumption on monocytes homeostasis, female and male mice received alcohol in drinking water per Meadows-Cook (MC) model, a well-accepted mouse model of chronic alcohol consumption to characterize immunological effects of alcohol (23, 24). We chose MC, not Lieber DeCarli (LD), the most common mouse model of chronic alcoholic liver injury, because we are interested in studying the specific effect of alcohol on immune cells without the interference of the high fat and liquid diet used in LD model. Moreover, previous work published by our group demonstrated that LD model shared with alcohol exposure alone only a very restricted number of altered pathways (24).

Four weeks of alcohol consumption caused a significant reduction in the body weight, but not liver weight, of male mice; however, normalized liver/body weight ratio, surrogate for steatosis development, showed a significant increase only in female mice compared to controls (**Supplementary Figures 1A–C**). Despite no significant changes in liver triglycerides and liver enzymes, histological examination showed very mild small droplet steatosis in only alcohol-fed female mice compared to their controls (**Supplementary Figures 1D, E** and **Supplementary Figures 2A–C**). Serum levels of LPS in alcohol-fed female mice increased but did not reach statistical significance when compared with female control mice (**Supplementary Figure 2D**). Also, after alcohol consumption, the serum level of LPS in female mice is higher than in male mice but without statistical significance (**Supplementary Figure 2D**). Hepatic leukocytes did not increase in alcohol-fed mice in females and males (**Supplementary Figure 3**). Interestingly, female mice have more hepatic leukocytes than males in only alcohol-fed mice (**Supplementary Figure 3**). The presence of small droplet discrete liver steatosis and increased liver/body weight ratio support early histological evidence of alcoholic liver injury in female mice, while the increase in number of hepatic leukocytes in alcohol-fed female mice compared to males may represent a potential modulator for susceptibility to alcoholic liver injury in females (19–21). We have shown that alcohol has a sex specific effect on innate immune cells after 3 months of alcohol exposure (25) so we wanted to further study innate immune cellular compartments affected during this earlier time points.

**Figure 1:**
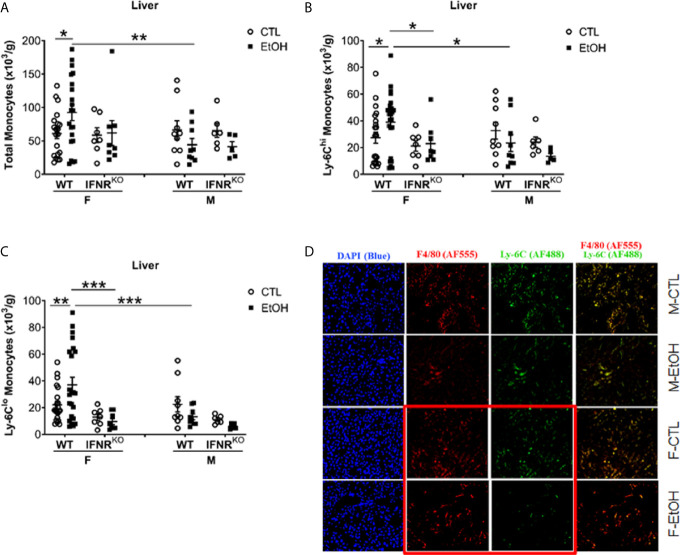
Alcohol induced increase in hepatic MDMs in female mice. Female (F) and male (M) From wild type (WT) and interferon α/β receptor 1-knockout (IFNR^KO^) mice were provided a regular chow diet and ethanol in drinking water (EtOH) or ethanol-free water (CTL). After 4 weeks, mice were euthanized, and Liver harvested. **(A–C)** Hepatic leukocytes were stained and analyzed by flow cytometry. Dot graphs showing the numbers of total monocytes **(A)**, Ly-6C^hi^ monocytes **(B)**, and Ly-6C^lo^ monocytes **(C)** per gram of liver weight. Values are showing the mean ± SEM, *p<0.05, **p<0.01, ***p<0.001. n ≥ 5. **(D)** Images are showing liver tissue stained with fluorescent-labeled antibodies, anti-F4/80 (AF555/red), anti-Ly-6C (AF488/green), and DAPI (blue).

Hepatic leukocytes were isolated and stained for flow cytometry analysis and gated to identify hepatic monocytes, neutrophils, and DCs (**Supplementary Figure 4**). Females that consumed alcohol for four weeks showed a significantly greater increase in hepatic monocytes than controls and alcohol-fed male mice (**Figure 1A**). Since these hepatic monocytes were isolated from perfused livers we will refer to them from here on as monocyte derived macrophages (MDM). However, we observed no statistically significant change in hepatic neutrophils and DCs (**Supplementary Figure 5**) between alcohol-fed and control mice in both female and male groups at this early time point. These data demonstrated that one of the earliest immunological innate events induced by alcohol in mice is identified in the liver at the level of monocytes compartment and present only in females. This sex dependent immunological effect of alcohol on liver monocytes combined with the well-known dichotomy in susceptibility of alcoholic immune effects triggered us to explore the mechanism of alcohol exposure on hepatic monocytes.

### Hepatic MDM Increase in Female Mice Is Due to Increase of Both LY-6C^hi^ and Ly-6C^lo^ Subsets

Classically in mice, monocytes are divided into two primary subsets based on the phenotypic expression of Ly-6C, CCR2, and CX3CR1 (1, 2). Monocytes expressing high levels of Ly-6C and CCR2 (Ly-6C^hi^CCR2^+^) rapidly migrate to sites of inflammation to give rise to inflammatory macrophages and DCs and are known as “inflammatory monocytes” (1, 2). The locally patrolling monocytes express high levels of CX3CR1 but low levels of both Ly-6C and CCR2 and described phenotypically as Ly-6C^lo^CX3CR1^+^ (1, 2). Investigating the expression of Ly-6C on hepatic monocytes revealed a significant increase only in Ly-6C^hi^ and Ly-6C^lo^ MDMs in alcohol-fed female mice compared to controls (**Figures 1B, C**). Also, in alcohol consumed mice, females have more Ly-6C^hi^ and Ly-6C^lo^ MDMs than males (**Figures 1B, C**). Additionally, histological examination using immune-fluorescent microscopy showed a reduction in Ly-6C staining in the liver tissue from female mice consumed alcohol and controls compared to their male counterparts (**Figure 1D**). These data indicate that the hepatic MDMs increase is the result of the expansion of both Ly-6C^hi^ and Ly-6C^lo^ subset numbers.

### Alcohol Consumption Induced a Reduction in the Serum Level of M-CSF but Did Not Alter the Numbers nor the Proliferation of BM Progenitors

**Figure 2:**
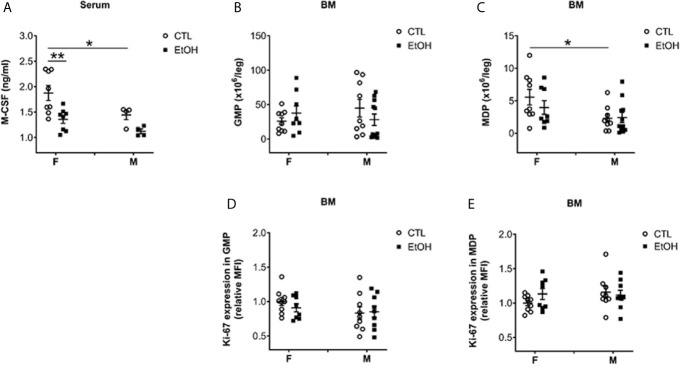
Alcohol consumption reduced M-CSF serum level in female mice but did not change the numbers nor the proliferation of classical BM progenitors. Female (F) and male (M) from wild type (WT) mice were provided a regular chow diet and ethanol in drinking water (EtOH) or ethanol-free water (CTL). After 4 weeks, mice were euthanized, and blood and BM collected. **(A)** Dot graphs showing the serum level of M-CSF. n ≥ 4. **(B–E)** BM cells counted then stained for flow cytometry analysis. BC) Dot graphs showing the numbers of GMP **(B)** and MDP **(C)** per leg. **(D, E)** Dot graphs showing Ki-67 expression (relative MFI) in GMP **(D)** and MDP **(E)**. n ≥ 8. Values are showing the mean ± SEM, *p<0.05, **p<0.01.

Monocyte/Macrophage colony-stimulating factor (M-CSF), a growth factor shown to be essential for monocyte homeostasis and differentiation (2, 26). Mice deficient in CD115, the M-CSF receptor, have deficiencies in monocytes and macrophages (2, 26). Therefore, we tested the serum level of M-CSF. Alcohol consumption induced a reduction in the serum level of M-CSF only in female mice compared to controls (**Figure 2A**). Additionally, control female mice showed higher levels of serum M-CSF compared to their male counterparts (**Figure 2A**). Studying BM leukocytes did not show a significant increase in alcohol-fed mice compared to controls (**Supplementary Figure 6**). Interestingly, there was a significant decrease in BM leukocytes in females compared to males in control groups (**Supplementary Figure 6**). Considering the increase in hepatic leukocytes in females compared to males (**Supplementary Figure 3**), these data might indicate an organ-specific nature of the potential immune-cellular mechanism(s) that might explain the higher susceptibility of females to develop alcoholic tissue injury. We studied the classically identified BM progenitors gated as shown in **Supplementary Figures 7** and **8**. The data did not reveal a change in granulocyte-macrophage progenitor (GMP) (**Figure 2B**) and macrophage-dendritic cell progenitor (MDP) (**Figure 2C**) in alcohol-fed mice compared to controls. Investigating the expression of Ki-67, a cell proliferation marker (27), in GMP and MDP did not reveal significant changes after alcohol consumption (**Figures 2D, E**). These data suggest that the increase in monocytes found in female mice is not a result of alcohol interference in the development of BM classical progenitors.

### Alcohol Consumption in Female Mice Induced Increase in the Expression of Hepatic TNFα

**Figure 3:**
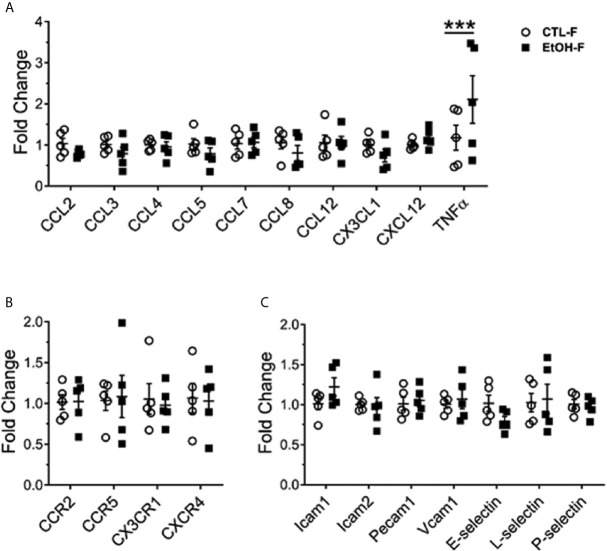
Increase in hepatic TNFα in alcohol-fed female mice. Female wild type mice were provided a regular chow diet and ethanol in drinking water (EtOH) or ethanol-free water (CTL). After 4 weeks, mice were euthanized, and liver harvested. Hepatic total mRNA extracted followed by qRT-PCR analysis of chemokines **(A)**, chemokine receptor **(B)**, and adhesion molecules **(C)** involved in monocyte recruitment. Dot graphs showing fold change in gene expression in alcohol-fed versus control mice calculated by comparative Ct method. n = 5. Values are showing the mean ± SEM ***p<0.001.

We asked whether the increase in hepatic MDMs results in a change of hepatic cytokine expression and whether this reflects a change in the hepatic chemokines and adhesion molecules gradient implicated in the recruitment and migration of monocytes (28). The cytokine tumor necrosis factor alpha (TNFα) was upregulated in the livers of female mice fed alcohol compared to their control counterparts (**Figure 3A**). The expression of chemokines, chemokine receptors, and adhesion molecules tested did not reveal significant changes between alcohol-fed and control female mice (**Figures 3A–C**). The increase in TNFα data is consistent with overwhelming evidence in the literature about the implications of TNFα in alcohol-induced liver injury in both humans and mice and marks an early event in alcohol immunopathology (29–33).

### Alcohol Consumption Did Not Increase Hepatic MDMs in IFNR^KO^ Mice as It Does in WT Mice

Our data did not reveal a disturbance in BM progenitors or recruitment and migration molecules that might explain the increase in MDMs we found in female mice that consumed alcohol. Published data showed regulation of emergent monocytopoiesis by type I interferon (IFN-I) signaling (34). Therefore, we used Interferon α/β receptor 1-deficient (IFNR^KO^) mice to study the effect of alcohol on hepatic monocytes in the absence of IFN-I signaling. Interestingly, IFNR^KO^ female mice consumed alcohol for four weeks failed to show an increase in their hepatic MDMs compared to control-fed mice, as did their WT counterparts (**Figures 1A–C**). In the livers of females, there are more Ly-6C^hi^ and Ly-6C^lo^ MDMs in WT compared to IFNR^KO^, in alcohol-fed mice (**Figures 1B, C**). This data suggest a critical role for IFN-I signaling in the increase of MDMs induced by alcohol in female mice.

### IFNR^KO^ Mice Have Fewer BM Monocyte Progenitors Than WT Mice

**Figure 4:**
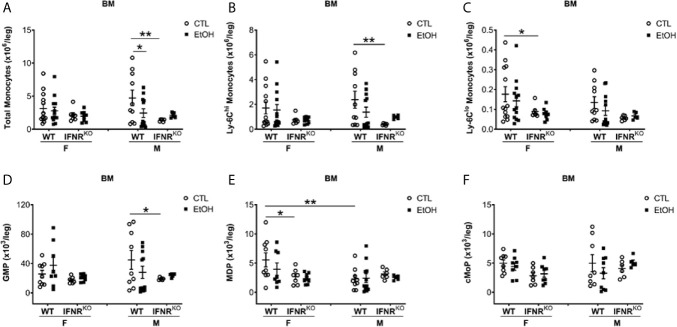
Reduction in MDP, a monocyte BM progenitor, in female IFNR^KO^ mice. Female (F) and male (M) from wild type (WT) and interferon α/β receptor knockout (IFNR^KO^) mice were provided a regular chow diet and ethanol in drinking water (EtOH) or ethanol-free water (CTL). After 4 weeks, mice were euthanized, and BM collected, cells counted, then stained for flow cytometry analysis. **(A–F)** Dot graphs showing the numbers per leg of total monocytes **(A)**, Ly-6C^hi^ monocytes **(B)**, Ly-6C^lo^ monocytes **(C)**, GMP **(D)**, MDP **(E)**, and cMoP **(F)**. n ≥ 5. Values showing the mean ± SEM, *p<0.05, **p<0.01.

We further investigated the BM development of monocytes in both WT and IFNR^KO^ mice. First, we investigated mature monocytes in the BM, and in both WT and IFNR^KO^ mice, our data suggest no change in monocyte subsets between alcohol-fed mice and controls (**Figures 4A–C**). Comparing BM progenitors between alcohol-fed and control mice did not show significant changes in both WT and IFNR^KO^ mice (**Figures 4D–F**). However, comparing the same progenitors between WT and IFNR^KO^ mice revealed a significant reduction in MDP in IFNR^KO^ female control mice (**Figure 4E**). In IFNR^KO^, both control and alcohol-fed female mice, compared to WT, there is a reduction in common monocyte progenitors (cMoP), but it did not reach statistical significance (**Figure 4F**). These data further demonstrate a potential role of IFN-I signaling in the increase of Ly-6C^hi^ and Ly-6C^lo^ subsets in female mice early during alcohol consumption and preceding histological findings of alcoholic liver injury.

## Discussion

Monocytes are innate immune cells recruited in sterile and pathogen induced inflamed tissues, critical in the clearance of pathogens and cellular debris as well as tissue return to steady-state condition (16). Multiple lines of evidence suggest the importance of monocytes in alcoholic tissue injury. Firstly, the elevation of neopterin and leukocyte-function-associated antigen 3, markers associated with monocyte activation, in patients with ALD (16, 35). Additionally, the expression of TNFα receptors and the spontaneous secretion of TNFα from circulating monocytes isolated from ALD patients and the association of high serum TNFα with poorer prognosis of acute alcoholic hepatitis patients all further suggests the critical role of monocytes in ALD (16). Furthermore, the migration of monocytes to the liver during inflammation and their conversion to a macrophage like phenotype, which play a critical role in eliminating pathogens and induce tissue repair (16, 17), indirectly further highlights the important role of monocytes in ALD.

In our experiments only very early and discrete signs of alcoholic liver injury are observed as increased small droplet steatosis without changes in liver triglycerides in alcohol-fed female mice after 4 weeks. This suggests rather early involvement of hepatocyte lipid transport and changes in non-triglyceride lipid fraction (sphingolipids, ceramides) by alcohol in female mice and less quantitative changes in triglycerides.

After alcohol consumption, female mice have higher leukocyte numbers compared with males and both, acute and chronic liver injuries, induce the recruitment of circulating monocytes into the liver (16). In our experiment, alcohol exposure increased hepatic MDMs in female mice after only four weeks. No significant changes were found in other recruited examined phagocytes, DCs and neutrophils. Upon further investigation, the increase in hepatic MDMs in female mice after four weeks of alcohol consumption was due to a rise in both Ly-6C^hi^ and Ly-6C^lo^ subsets. The increase in hepatic MDM subsets is consistent with upregulation of TNFα mRNA as macrophages are known to produce it (16). It was reported that Ly-6C^hi^ monocytes recruited following liver injury then undergo a phenotype switch to become Ly-6C^lo^ monocytes within few days (5, 36, 37). Zigmond et al. showed in male mice, that liver injury induced by N-acetyl-p-aminophenol led to the recruitment of Ly-6C^hi^ monocytes, which then differentiate into Ly-6C^lo^ monocyte derived macrophages (36). Dal-Secco *et al.* showed that in sterile liver injury, Ly-6C^hi^ (CCR2^hi^ CX3CR1^lo^) monocytes migrated to the injured area then transitioned to Ly-6C^lo^ (CCR2^lo^ CX3CR1^hi^) monocytes (37). The data presented by both studies could support the concept that the increase in hepatic Ly-6C^hi^ and Ly-6C^lo^ in female mice after four weeks of alcohol consumption may be due to increased recruitment of Ly-6C^hi^ then their conversion to Ly-6C^lo^ MDMs. However, at this time, we cannot rule out the alternative hypothesis that the accumulation of hepatic Ly-6C^lo^ MDMs may be, at least in part, due to increased hepatic recruitment or survival of Ly-6C^lo^ MDMs.

To investigate the mechanism responsible for the early expansion of hepatic MDMs after alcohol exposure in female mice, we studied monocyte development. M-CSF is an important growth factor for the homeostasis and differentiation of monocytes (2, 26) and was found to be suppressed by alcohol. The reduction in the serum levels of M-CSF in alcohol-fed mice compared to controls in females is inconsistent with the reports on the non-alcoholic chronic liver disease where hepatic inflammation in female and male humans is associated with an increase in serum M-CSF (38, 39). However, our data is consistent with the observation that women have higher serum concentrations of M-CSF than men (40). The reduction of serum M-CSF in alcohol-fed female mice, suggests of a possible negative feedback effect on monocytopoiesis or direct alcohol effect on M-CSF sources. Studies on the effect of alcohol on M-CSF are scarce. Kottstorfer *et al.* found no significant correlation in humans between alcohol consumption and the expression of M-CSF (40). In spite of this suppressive effect of alcohol on circulating M-CSF levels, M-CSF levels in females are still significantly higher than males, which could potentially additionally explain the consistent increase in monocytes found in female mice after alcohol consumption. Analyzing classical BM progenitors such as GMP and MDP (2), we did not reveal significant changes in the bone marrow that could explain the increase in monocytes. Consistent with that, alcohol consumption for three months did not alter GMP progenitors in a Rhesus Monkey study (41).

Monocytes originate in the BM under the control of the growth factor M-CSF and circulate in the blood, and do not proliferate during steady state (26, 42). Monocyte development, monocytopoiesis, is regulated by the expression or suppression of transcription factors such as Transcription factor PU.1, Interferon regulatory factor-8, and Kruppel-like factor 4 (2). Induction of monocytopoiesis by IFN-I signaling during endotoxemia has been shown in the literature (34). Consistent with that, our data revealed the failure of IFNR^KO^ mice exposed to alcohol to increase their hepatic Ly-6C^hi^ and Ly-6C^lo^ MDM subsets as did their WT counterparts. The data from the IFNR^KO^ mice, compared to WT mice, also showed a reduction in mature Ly-6C^lo^ monocytes in both liver and BM. In an acute model of sterile inflammation using a murine model of pristane-induced peritonitis the IFN-I signaling was responsible for monocyte recruitment and maturation during inflammation while mice deficient in TLR4, TNFα, IL-6, and IL1R failed to accumulate monocytes in the peritoneal cavity (43). This is consistent with our data that IFN-I signaling is critical for increasing hepatic MDMs upon alcohol consumption. However, in our case, the increase was in both Ly-6C^hi^ and Ly-6C^lo^ subsets compared to the Ly-6C^hi^ subset in the first phase of acute tissue injury model. In a second phase, the accumulation of Ly-6C^hi^ monocytes was replaced by Ly-6C^lo^ monocytes within 72 hours from the challenge, consistent with our finding (43).

MDP precursors and cMoP precursors in the BM are macrophage/monocyte progenitors (44). Studying MDP and cMoP progenitors in the BM of IFNR^KO^ mice compared to WT revealed a reduction in MDP progenitors in female control mice, suggesting the importance of IFN-I signaling to maintain MDP progenitors at least during steady state. Lasseaux *et al.* reported a reduction in cMoP progenitors in mice 24 hours after intravenous injection with LPS, the sex of mice used in the experiments is not indicated (34), which is inconsistent with our data revealed no change in cMoP after alcohol consumption. This could be due to the fact that they used LPS compared to alcohol in our study. Interestingly, IFNR^KO^ mice did not increase monocyte precursors after the LPS challenge, although the mice showed the reduction of cMoP progenitors (34), suggesting the IFN-I dependency of the monocytopoiesis during inflammation consistent with our data. It is important to state that more prolonged exposure to alcohol for 3 months in the same model in female mice expanded hepatic plasmacytoid DCs (25); whether that is an earlier event represented by bone marrow pDC expansion and paracrine source of IFNα for stimulation of monocytopoiesis by alcohol is under ongoing evaluation in our laboratory.

In summary, our data strongly suggest that alcohol exposure for four weeks in mice induces an increase of Ly-6C^hi^ and Ly-6C^lo^ MDMs in an IFN-I signaling-dependent manner only in female mice. Our data support the need for additional investigations of the cellular sources of IFNα and functional relevance of the hepatic MDM subsets expansion in female mice early upon alcohol exposure as a mechanism for increased susceptibility to alcoholic immunopathogenesis.

## Data Availability Statement

The original contributions presented in the study are included in the article/**Supplementary Material**. Further inquiries can be directed to the corresponding author.

## Ethics Statement

The animal study was reviewed and approved by Rush University Medical Center, Institutional Animal Care & Use Committee.

## Author Contributions

KA and HF conduct the experiments, analyzed the data, and wrote the manuscript. AV and TK conduct the experiments. MK conduct the experiment, analyzed the data, and reviewed the manuscript. CA obtained the funding, designed the project, analyzed the data, and edited and reviewed the manuscript. All authors contributed to the article and approved the submitted version.

## Funding

This work is supported by grant from the National Institutes of Health: AA024762 awarded CA.

## Conflict of Interest

The authors declare that the research was conducted in the absence of any commercial or financial relationships that could be construed as a potential conflict of interest.

## Abbreviations

ALD, Alcoholic liver disease; BM, Bone marrow; CCR2, C-C motif chemokine receptor 2; cMoP, Common monocyte progenitor; CX3CR1, C-X3-C motif chemokine receptor 1; DC, Dendritic cells; EtOH, Alcohol (ethanol); GMP, Granulocyte-macrophage progenitor; HE, Hematoxylin and Eosin; IFN-I, Type 1 interferon; IFNR^KO^, Interferon α/β receptor 1-knockout; KC, Kupffer cells; LD, Lieber DeCarli model; LPS, Lipopolysaccharide; Ly-6C, Lymphocyte antigen 6 complex, locus C1; MC, Meadows-Cook model; M-CSF, Monocyte/Macrophage colony-stimulating factor; MDM, Monocyte-derived macrophages; MDP, Macrophage-dendritic cell progenitor; TNFα, tumor necrosis factor alpha; WT, Wild type.
