# Supplementary material for: Alcohol Consumption Accumulation of Monocyte Derived Macrophages in Female Mice Liver Is Interferon Alpha Receptor Dependent
Source: Front Immunol. 2021 Apr 30;12:663548. doi: 10.3389/fimmu.2021.663548 (PMC8119877; doi:10.3389/fimmu.2021.663548)
Supplement: Supplementary file 1 [file DataSheet_1.pdf]

Supplementary Table 1. Fluorophores-conjugated antibodies used for flow cytometry analysis

Antibody	Clone	Fluorophore	Vender
CD16/32	93	APC-Cy7	BioLegend
CD45	30-F11	APC-Cy7	BioLegend
CD45R/B220	RA3-6B2	BV711	BD Bioscience
NK1.1	PK136	PE-CF594	BD Bioscience
NK1.1	PK136	BV605	BD Bioscience
CD3	145-2C11	PE-CF594	BD Bioscience
CD3	145-2C11	PE	BD Bioscience
CD19	1D3	PE-CF594	BD Bioscience
Ly-6C	HK1.4	BV785	BioLegend
Ly-6G	1A8	Alexa Fluor 700	BD Bioscience
PDCA-1	927	FITC	BioLegend
MHC-II (I-A/I-E)	M5/114.15.2	PE-Cy7	BioLegend
CD11b	M1/70	BV421	BD Bioscience
CD11b	M1/70	PE-CF594	BD Bioscience
CD11c	N418	BV650	BioLegend
CD115	AFS98	APC	BioLegend
CD115	AFS98	Alexa Fluor 488	BioLegend
CD117	2B8	PE-Cy7	BioLegend
CD117	2B8	BV650	BioLegend
CD135	A2F10	PE-Cy5	BioLegend
F4/80	BM8	APC	BioLegend
Gr-1	RB6-8C5	PE-CF594	BD Bioscience
Gr-1	RB6-8C5	Pacific Blue	BioLegend
Ki-67	SolA15	PE	Invitrogen
Sca-1	D7	FITC	eBioscience
Sca-1	D7	Alexa Fluor 700	eBioscience
Ter119	TER-119	PerCP-Cy5.5	BioLegend
