# Supplementary material for: Alcohol Consumption Accumulation of Monocyte Derived Macrophages in Female Mice Liver Is Interferon Alpha Receptor Dependent
Source: Front Immunol. 2021 Apr 30;12:663548. doi: 10.3389/fimmu.2021.663548 (PMC8119877; doi:10.3389/fimmu.2021.663548)
Supplement: Supplementary file 2 [file DataSheet_2.pdf]

Supplementary Table 2. Flow cytometry gating and identification of different cell populations

Cell name	Phenotype (All cell were gated on live single cells)
Leukocytes	$CD45^{pos}$
Neutrophils	$CD45^{pos}Lin(CD3/CD19/NK1.1)^{neg}Ly\text{-}6G^{pos}PDCA\text{-}1^{neg}CD11b^{pos}Ly\text{-}6C^{pos}$
Dendritic cells	$CD45^{pos}Lin(CD3/CD19/NK1.1)^{neg}Ly\text{-}6G^{neg}PDCA\text{-}1^{neg}CD11c^{hi}MHCII^{hi}$
Total monocytes	$CD45^{pos}Lin(CD3/CD19/NK1.1)^{neg}Ly\text{-}6G^{neg}PDCA\text{-}1^{neg}MHCII^{neg}CD11b^{pos}F4/80^{pos}$
$Ly\text{-}6C^{hi}$ monocytes	$CD45^{pos}Lin(CD3/CD19/NK1.1)^{neg}Ly\text{-}6G^{neg}PDCA\text{-}1^{neg}MHCII^{neg}CD11b^{pos}F4/80^{pos}Ly\text{-}6C^{hi}$
$Ly\text{-}6C^{lo}$ monocytes	$CD45^{pos}Lin(CD3/CD19/NK1.1)^{neg}Ly\text{-}6G^{neg}PDCA\text{-}1^{neg}MHCII^{neg}CD11b^{pos}F4/80^{pos}Ly\text{-}6C^{lo}$
GMP progenitors	$Gr1^{neg}B220^{neg}Sca1^{neg}CD11c^{neg}CD3^{neg}NK1.1^{neg}CD11b^{neg}Ter119^{neg}CD16/32^{pos}CD135^{pos}CD115^{neg}CD117^{pos}$
MDP progenitors	$Gr1^{neg}B220^{neg}Sca1^{neg}CD11c^{neg}CD3^{neg}NK1.1^{neg}CD11b^{neg}Ter119^{neg}CD16/32^{pos}CD135^{pos}CD115^{pos}CD117^{pos}$
cMoP progenitors	$Gr1^{neg}B220^{neg}Sca1^{neg}CD11c^{neg}CD3^{neg}NK1.1^{neg}CD11b^{neg}Ter119^{neg}CD117^{pos}CD135^{neg}CD115^{pos}$
