# Supplementary material for: Alcohol Consumption Accumulation of Monocyte Derived Macrophages in Female Mice Liver Is Interferon Alpha Receptor Dependent
Source: Front Immunol. 2021 Apr 30;12:663548. doi: 10.3389/fimmu.2021.663548 (PMC8119877; doi:10.3389/fimmu.2021.663548)
Supplement: Supplementary file 3 [file DataSheet_3.pdf]

Supplementary Table 3. List of primers used for qRT-PCR

Gene Name (Mouse)	Primer ID	Forward Primers	Reverse Primers
Ccl2	Mm00441242_m1	TAAAAACCTGGATCGGAACCAAA	GCATTAGCTTCAGATTTACGGGT
Ccl3	Mm00441259_g1	TGTACCATGACACTCTGCAAC	CAACGATGAATTGGCGTGGAA
Ccl4	Mm00443111_m1	TTCCTGCTGTTTCTCTTACACCT	CTGTCTGCCTCTTTTGGTCAG
Ccl7	Mm00443113_m1	GCTGCTTTCAGCATCCAAGTG	CCAGGGACACCGACTACTG
Ccl8	Mm01297183_m1	TCTACGCAGTGCTTCTTTGCC	AAGGGGGATCTTCAGCTTTAGTA
Ccl12	Mm01617100_m1	ATTTCCACACTTCTATGCCTCCT	ATCCAGTATGGTCCTGAAGATCA
Cx3cl1	Mm00436454_m1	CTGGCCGCGTTCTTCCATT	GCACATGATTTCGCATTTCGT
Cxcl12	Mm00445553_m1	TGCATCAGTGACGGTAAACCA	CACAGTTTGGAGTGTTGAGGAT
Icam1	Mm00516023_m1	TGCCTCTGAAGCTCGGATATAC	TCTGTCGAACTCCTCAGTCAC
Icam2	Mm00494862_m1	GCAGGACAACCAAATGGTCAT	AGAACAGCAGTATTGACACCAC
Pecam1	Mm01242576_m1	ACGCTGGTGCTCTATGCAAG	TCAGTTGCTGCCCATTCATCA
Vcam1	Mm01320970_m1	TTCGGTTGTTCTGACGTGTG	TACCACCCCATTGAGGGGAC
E-selectin	Mm00441278_m1	ATGAAGCCAGTGCATACTGTC	CGGTGAATGTTTCAGATTGGAGT
L-selectin	Mm00441291_m1	TACATTGCCCAAAAGCCCTTAT	CCTCCTTGGACTTCTTGTTGTT
P-selectin	Mm01204601_m1	CCACCGAAGTCCCTTCCAC	GCCGCTGTCAGGTAAGTGA
Actb	Mm02619580_g1	ACCCGCCTCACATTGAAATCC	GGCGTATGTATCAGTCTCAGTG
B2m	Mm00437762_m1	TTCTGGTGCTTGTCTCACTGA	CAGTATGTTCGGCTTCCCATTC
Ccl5	Mm.PT.58.43548565	GCTCCAATCTTGCAGTCGT	CCTCTATCCTAGCTCATCTCCA
Tnfa	Mm.PT.58.12575861	AGACCCTCACACTCAGATCA	TCTTTGAGATCCATGCCGTTG
B2m	Mm.PT.39a.22214835	TGGTCTTTCTGGTGCTTGTC	GGGTGGAACTGTGTTACGTAG
