# Supplementary material for: Alcohol Consumption Accumulation of Monocyte Derived Macrophages in Female Mice Liver Is Interferon Alpha Receptor Dependent
Source: Front Immunol. 2021 Apr 30;12:663548. doi: 10.3389/fimmu.2021.663548 (PMC8119877; doi:10.3389/fimmu.2021.663548)
Supplement: Supplementary file 4 [file DataSheet_4.pdf]

Supplementary Figure 1

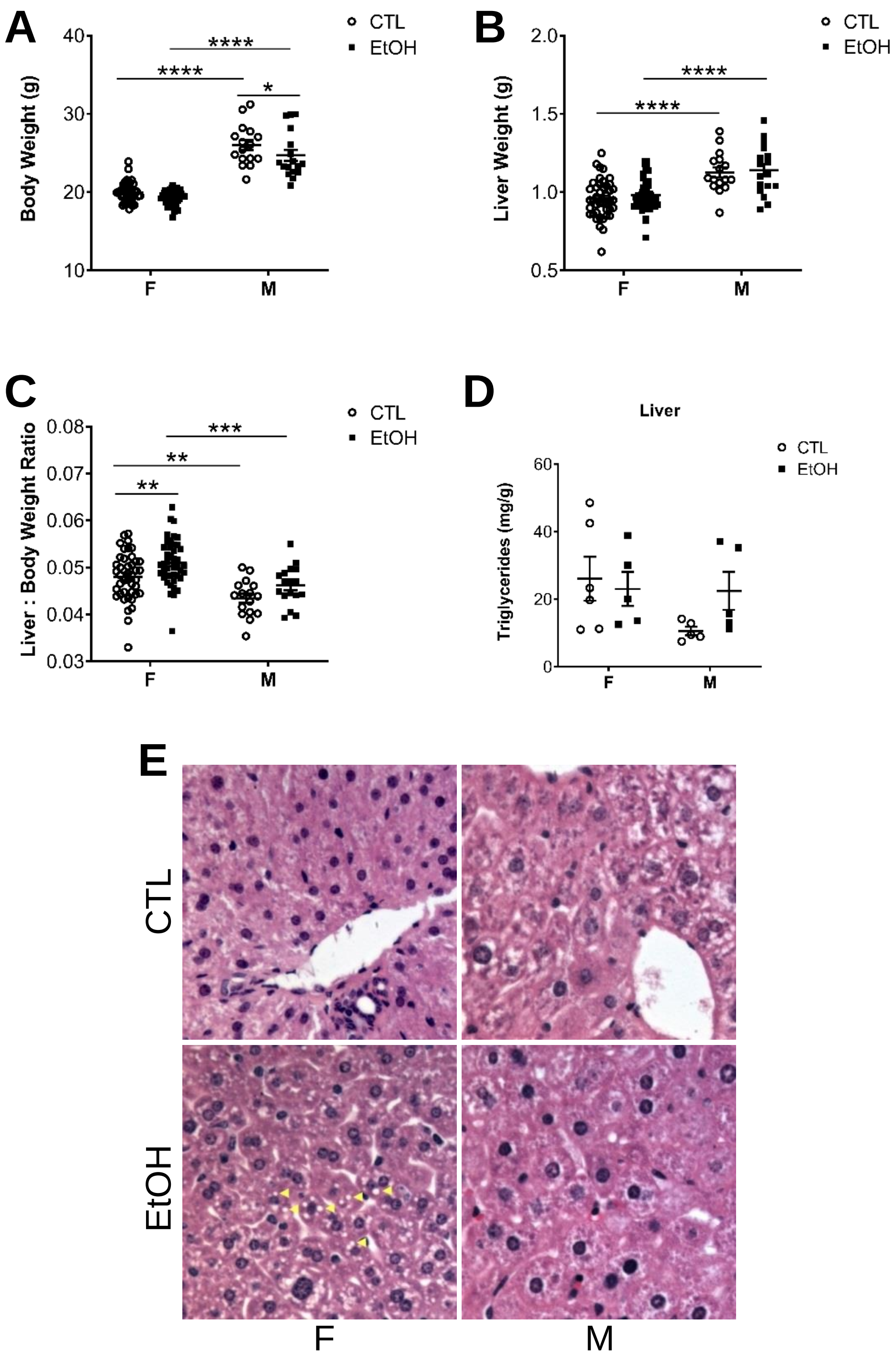

Supplementary Figure 1. Four weeks of alcohol consumption induced mild steatosis in female mice. Female (F) and male (M) mice provided a regular chow diet and ethanol in drinking water (EtOH) or ethanol-free water (CTL). After 4 weeks, mice euthanized, and body and liver weight measured. The livers were processed for histological analysis and triglycerides measurement. Dot graphs showing body weight (A), liver weight (B), and liver/body weight ratio (C). n ≥ 16. D) Dot graphs showing liver triglycerides. n ≥ 5. Values are showing the mean ±SEM, **p<0.01, ***p<0.001, ****p<0.0001. E) Images are showing liver tissue stained with Hematoxylin and Eosin.

Supplementary Figure 2

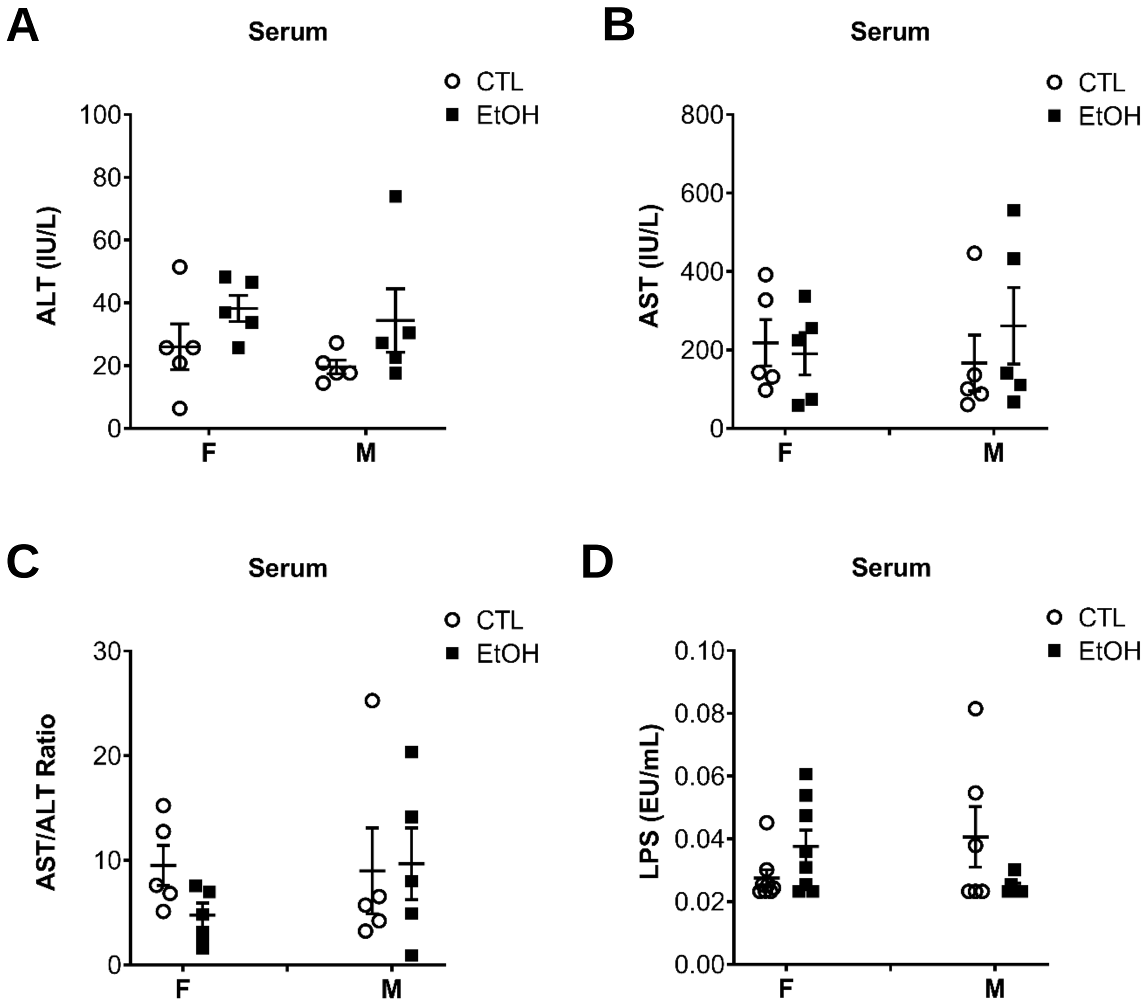

Supplementary Figure 2. No significant change in the serum levels of liver enzymes and LPS between alcohol-fed and control mice. Female (F) and male (M) mice provided a regular chow diet and ethanol in drinking water (EtOH) or ethanol-free water (CTL). After 4 weeks, mice euthanized, and blood was collected. Dot graphs showing ALT (A), AST (B), AST/ALT ratio (C), and LPS (D) levels. n ≥ 5. Values are showing the mean ±SEM.

Supplementary Figure 3

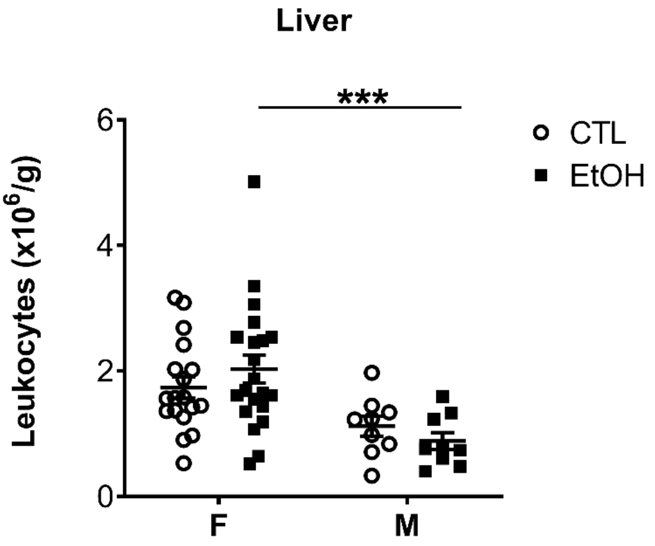

Supplementary Figure 3. Alcohol consumption did not alter the total intrahepatic leukocytes numbers. Female (F) and male (M) mice provided a regular chow diet and ethanol in drinking water (EtOH) or ethanol-free water (CTL). After 4 weeks, mice euthanized, and intrahepatic leukocytes were isolated and counted then stained for flow cytometry analysis. $n \geq 9$. Values are showing the mean ±SEM, ***$p<0.001$.

Supplementary Figure 4

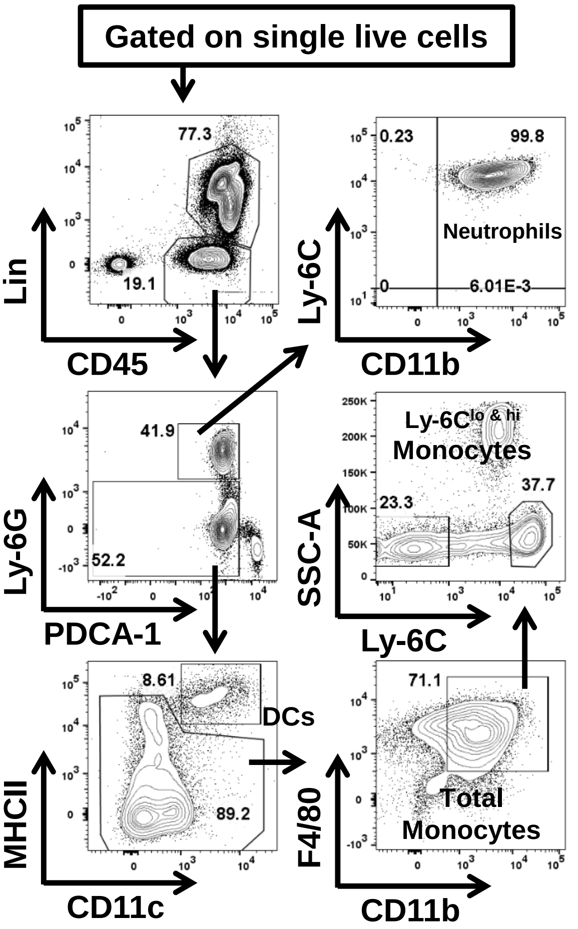

Supplementary Figure 4. Gating strategy for the identification of myeloid cells. Representative dot plots showing the gating strategy used to identify monocytes, neutrophils, and dendritic cells. Cells gated on single live cells. Monocytes are identified as: $CD45^{pos}Lin(CD3/CD19/NK1.1)^{neg}Ly\text{-}6G^{neg}PDCA\text{-}1^{neg}CD11c^{neg/pos}MHCII^{neg/pos}CD11b^{pos}F4/80^{pos}$. Neutrophils as: $CD45^{pos}Lin^{neg}Ly\text{-}6G^{pos}PDCA\text{-}1^{neg}CD11b^{pos}Ly\text{-}6C^{pos}$. Dendritic cells as: $CD45^{pos}Lin^{neg}Ly\text{-}6G^{neg}PDCA\text{-}1^{neg}CD11c^{hi}MHCII^{hi}$. The number inside the plots represent the percentages of the gated population.

Supplementary Figure 5

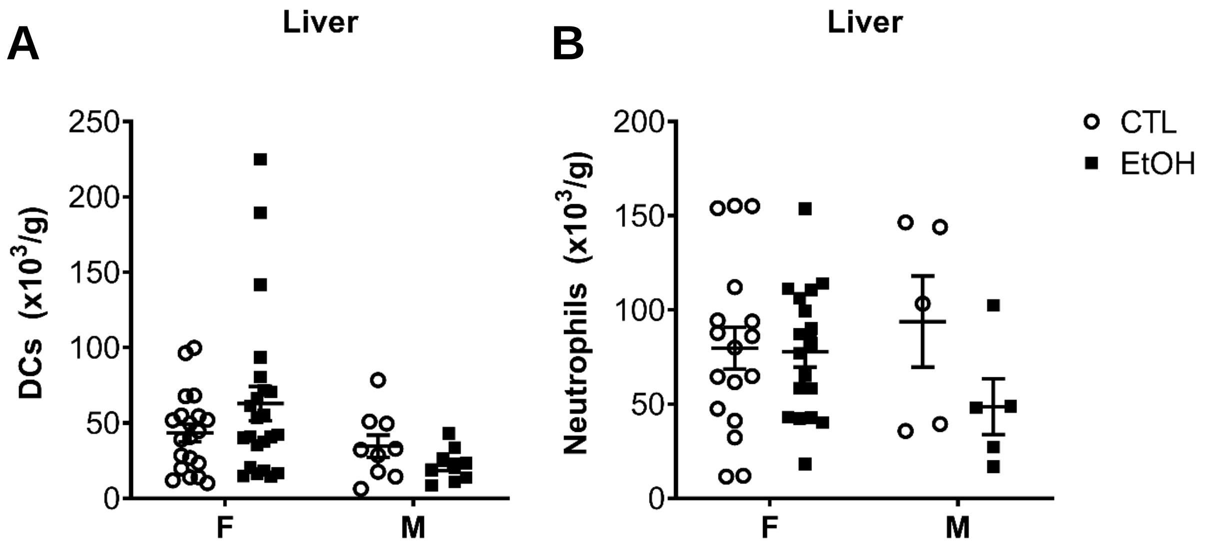

Supplementary Figure 5. No significant change in hepatic dendritic cells or neutrophils after 4 weeks of alcohol consumption. Female (F) and male (M) mice provided a regular chow diet and ethanol in drinking water (EtOH) or ethanol-free water (CTL). After 4 weeks, mice euthanized, and intrahepatic leukocytes were isolated and counted, then stained for flow cytometry analysis. Dot graph showing the numbers of DCs (A) and neutrophils (B) per gram of liver weight. $n \geq 5$. Values are showing the mean ±SEM.

Supplementary Figure 6

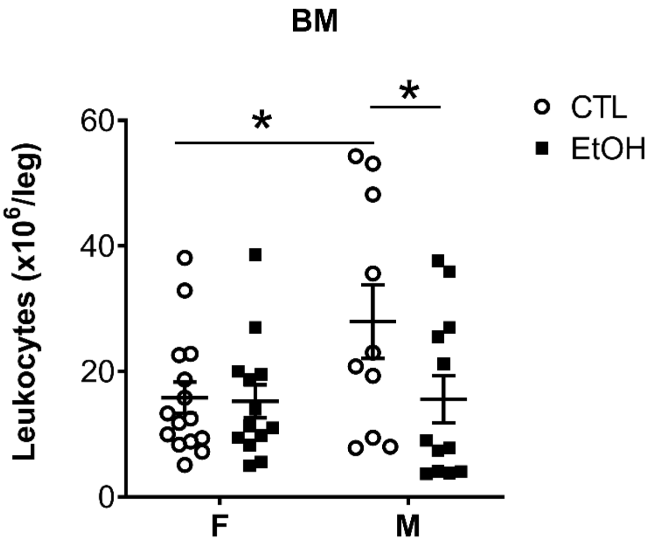

Supplementary Figure 6. No changes in BM leukocytes after alcohol consumption. Female (F) and male (M) mice provided a regular chow diet and ethanol in drinking water (EtOH) or ethanol-free water (CTL). After 4 weeks, mice euthanized, BM collected, and cells counted, then stained for flow cytometry analysis. Dot graph showing the numbers of leukocytes per leg. $n \geq 10$. Values are showing the mean ±SEM , *$p<0.05$.

Supplementary Figure 7

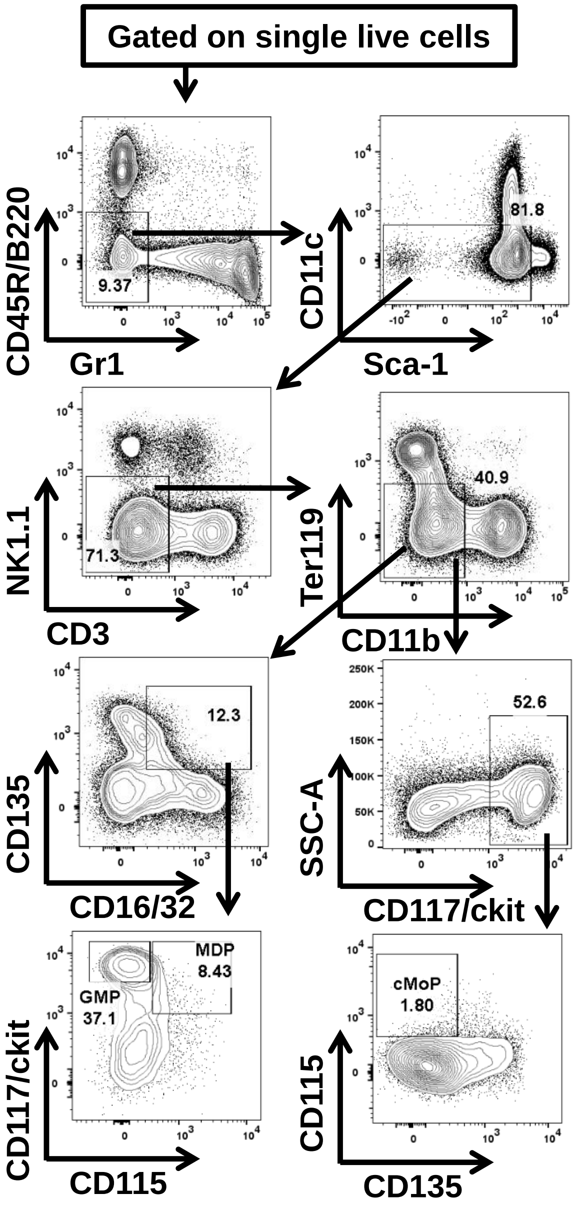

Supplementary Figure 7: The gating strategy for the identification of BM progenitors. Representative dot plots showing the gating strategy to identify the BM progenitors. GMP and MDP both identified as: Gr1^{neg}B220^{neg}Sca1^{neg}CD11c^{neg}CD3^{neg}NK1.1^{neg}CD11b^{neg}Ter119^{neg}CD16/32^{pos}CD135^{pos}, then GMP as: CD115^{neg}CD117^{pos} and MDP as CD115^{pos}CD117^{pos} . The cMoP is identified as: Gr1^{neg}B220^{neg}Sca1^{neg}CD11c^{neg}CD3^{neg}NK1.1^{neg}CD11b^{neg}Ter119^{neg}CD117^{pos}CD135^{neg}CD115^{pos}. The number inside the plots represent the percentages of the gated population

Supplementary Figure 8

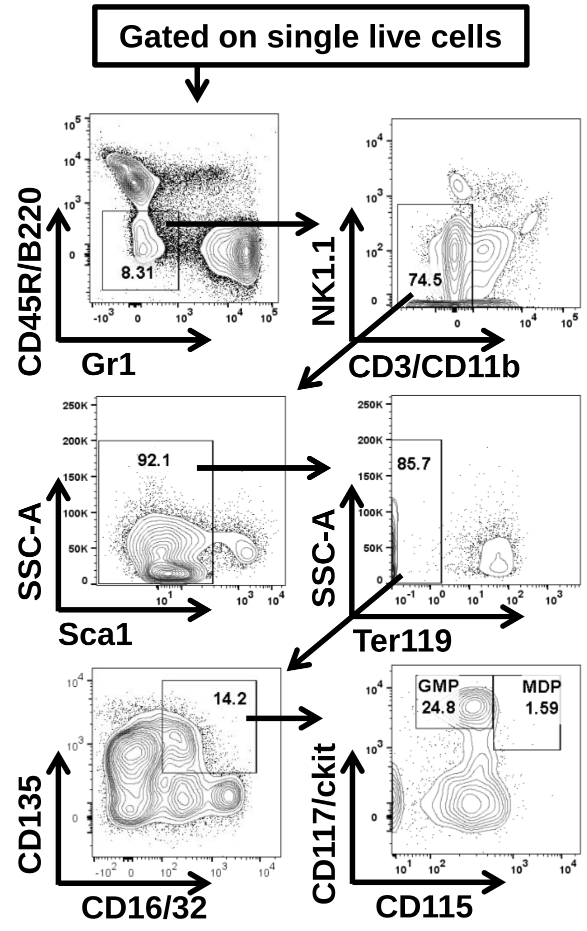

Supplementary Figure 8: The gating strategy for the identification of BM progenitors for Ki-67 staining. Representative dot plots showing the gating strategy to identify the BM progenitors. GMP and MDP both identified as: Gr1^{neg}B220^{neg}CD3^{neg}CD11b^{neg}NK1.1^{neg}Sca1^{neg}Ter119^{neg}CD16/32^{pos}CD135^{pos}, then GMP as: CD115^{neg}CD117^{pos} and MDP as CD115^{pos}CD117^{pos} . The number inside the plots represent the percentages of the gated population
